# Follie a deux: Psychopathology in a pandemic

**DOI:** 10.1192/j.eurpsy.2021.1434

**Published:** 2021-08-13

**Authors:** I. Soares Da Costa, A. Vieira, A. Amaral, F. Coutinho

**Affiliations:** Psychiatry And Mental Health Department, Centro Hospitalar e Universitário de São João, Porto, Portugal

**Keywords:** psychosis, psychopathology, follie a deux, pandemics

## Abstract

**Introduction:**

Follie a deux is a rare syndrome characterized by the transference of delusions from a primary subject to a secondary one. This rare condition, and frequently forgotten in psychiatry pratice, is more frequent in feedlots, particularly in situations alike we face nowadays because of the pandemic.

**Objectives:**

To describe a clinical case and to discuss and highlight some clinical aspects of this entity.

**Methods:**

Present a clinical case report and respective non systematic literature review

**Results:**

This clinical vignete describes a case of shared delusion between a mother and a son. The son suffers from an intelectual disability and shared with his mother a persecutory and prejudice delusion. Both live in the same house and because of the pandemic they spend all the time together. This situation was probably the main factor influencing the course of the symptoms.

**Conclusions:**

It is highlighted the importance of a social isolation and close contact between the pair mother/son, more important in context of a global pandemic, viewed as an obstacle to promote the separation of both.

